# Risk of mortality in the elderly with different degree of sensorineural hearing loss in Taiwan

**DOI:** 10.3389/fneur.2025.1628976

**Published:** 2025-11-05

**Authors:** Jin-Cherng Chen, Pei-Shan Hsieh, Juen-Haur Hwang

**Affiliations:** ^1^Department of Neurosurgery, Dalin Tzu Chi Hospital, Buddhist Tzu Chi Medical Foundation, Chiayi, Taiwan; ^2^School of Medicine, Tzu Chi University, Hualien, Taiwan; ^3^Department of Medical Research, Dalin Tzu Chi Hospital, Buddhist Tzu Chi Medical Foundation, Chiayi, Taiwan; ^4^Deparments of Otolaryngology-Head and Neck Surgery, Dalin Tzu Chi Hospital, Buddhist Tzu Chi Medical Foundation, Chiayi, Taiwan; ^5^Deparments of Otolaryngology-Head and Neck Surgery, Taichung Tzu Chi Hospital, Buddhist Tzu Chi Medical Foundation, Taichung, Taiwan; ^6^Department of Medical Research, China Medical University Hospital, China Medical University, Taichung, Taiwan

**Keywords:** mortality, age-related hearing impairment, elderly, sensorineural hearing loss, hearing level

## Abstract

**Introduction:**

Sensorineural hearing loss (SNHL) may lead to disability in many aspects. This study aims to investigate the risk of mortality in the elderly with sensorineural hearing loss (SNHL) in Taiwan.

**Methods:**

Three hundred and eighteen subjects with SNHL of age between 51 and 88 years old were included between August 2000 and December 2002. Averaged pure tone threshold of all tested six frequencies (250 Hz, 500 Hz, 1,000 Hz, 2000 Hz, 4,000 Hz, and 8,000 Hz) of both ears with all audiogram shapes was divided into three cohorts: normal hearing group [0–24 decibel hearing level (dBHL)]; mild SNHL group (25–39 dBHL); moderate and severe SNHL group (40–89 dBHL). The incidence rates of mortality were compared using the Kaplan–Meier method with the log-rank test. Association of SNHL and mortality was examined by a Cox proportional hazard model with adjustment for all covariates.

**Results:**

Compared to the normal hearing group, the crude hazard ratio (HR) and adjusted hazard ratio (aHR) for mortality in mild SNHL group was 1.51 (95% CI = 0.44–5.14, *p* = 0.5143) and 2.14 (95% CI = 0.45–10.23, *p* = 0.3396), respectively. And, the crude HR and aHR for mortality in moderate and severe SNHL group was 4.82 (95% CI = 1.65–14.07, *p* = 0.0041) and 6.96 (95% CI = 1.60–30.23, *p* = 0.0096), respectively.

**Conclusion:**

The risk of mortality was significantly higher in the elderly with moderate and severe SNHL.

## 1 Introduction

Sensorineural hearing loss (SNHL) may lead to disability in many aspects. Till now, only limited studies had discussed the relationship between SNHL and mortality in adults. Compared to normal-hearing control, subjects with severe [adjusted hazard ratio (aHR) = 4.07] or profound hearing loss (aHR = 4.22) were associated with a significant increase in mortality in Korean population who were older than 40 years old after adjusting for age, sex, region of residence, income, and past histories (1). They found that, compared with control group, the severe and profound hearing loss groups had higher mortality due to infection, metabolic, mental, circulatory, respiratory and digestive diseases, neoplasm, and trauma (1).

Compared with normal hearing group, subjects with hearing impairment were related with increased risks for total mortality and mortality from heart disease in American adults (2). The HRs for total mortality were 1.16, 1.54, and 1.85 for mild, moderate, and severe speech-frequency hearing loss, respectively. In addition, moderate SNHL (HR = 1.90) and severe SNHL (HR = 3.50) were significantly related with elevated risk of heart disease mortality, respectively (2). Compared with normal hearing group, those who had hearing problems (a little trouble-HR: 1.17; a lot of trouble-HR: 1.45; deaf-HR: 1.54, respectively) were at an increased risk of mortality from all causes and cancers in American adults (3). However, Zhang et al. (4) reported that hearing impairment was not significantly associated with all-cause mortality risk in elderly Chinese subjects, adjusting for living area, life style, and medical conditions.

Although some studies had suggested that SNHL might be associated with increased mortality in older adults, but their relationship was still inconsistent. The weak point or inconsistency between previous studies might be caused by different study population, imperfect classification of hearing impairment, and/or statistical strategy. In fact, SNHL is a disease which was caused by many genetic and environmental variations (5, 6). For example, obesity, hypertension, diabetes, dyslipidemia, coronary artery diseases, obstructive sleep apnea syndrome, transient ischemic attack might lead to SNHL (6–10). Thus, SNHL could be an intermediate indicator between many major diseases and mortality. Furthermore, SNHL itself might increase disability and the risks for cognition impairment, accident, depression, or stroke in the elderly (11–13). So, we supposed that SNHL could also increase the risk of mortality in the elderly independently.

Based on above introduction and criticism, we knew that the relationship between SHNL and mortality might be variable by ethnic groups and/or genetic background, and statistical strategy. Fortunately, compared to other nations, genetic heterogeneity was relatively low in Taiwan (5, 6), which could reduce the violation the clinical revelence of SNHL on all-cause mortality no matter how it contributed dependently or independently. In this study, we aimed to clarify this question by a cohort study using real world data of the elderly in Taiwan and a new statistical strategy in this study.

## 2 Materials and methods

To investigate whether SNHL might increase the risk of mortality in the elderly. We identified 318 subjects with symmetric SNHL of age between 51 and 88 years at the outpatient department between August 2000 and December 2002. The date of having the first result of audiometry with all audiogram shapes was considered the index date (14).

Averaged pure tone threshold of all tested six frequencies (250 Hz, 500 Hz, 1,000 Hz, 2000 Hz, 4,000 Hz and 800 Hz) of both ears was divided into three cohorts: normal hearing group [0–24 decibel hearing level (dBHL)]; mild hearing-impaired group (25–39 dBHL); moderate and severe hearing-impaired group (40–89 dBHL) (15). In addition, age, gender, body mass index (BMI), hypertension, diabetes mellitus (DM), dysplipidemia, coronary artery disease (CAD), acute myocardial infarction (AMI), chronic kidney disease (CKD), hepatitis, cirrhosis, Parkinson’s disease, Alzheimer’s disease, stroke, cancers, and trauma were recorded and will be adjusted during statistical analysis.

Patients who had conductive hearing impairment (averaged air-bone gap of hearing threshold greater than 10 dB HL), mixed type hearing impairment, and asymmetric SNHL (averaged pure tone threshold greater than 15 dB HL between both ears) before the index date were excluded.

### 2.1 Measurement of main outcome

Three cohorts were ended until the occurrence of death, or the July of 2011. That is, all cohorts were followed up to 10 years. All causes of death were recorded and included.

### 2.2 Ethical considerations

The institutional review board of Dalin Tzu Chi Hospital, Buddhist Tzu Chi Medical Foundation in Taiwan has approved this study. The informed consent of all patients was waived due to de-identified data during analysis (No. B10202021).

### 2.3 Statistical analyses

Categorical and continuous variables were compared by Pearson’s chi-squared test and one-way ANOVA test, respectively. The incidence rates (per 10^2^ person-years) with 95% confidence intervals (CIs) of all-cause mortality were calculated by Kaplan–Meier method and the log-rank test.

The association of SNHL and mortality was checked by a Cox proportional hazard model with adjustment for all covariates. As we introduced, SNHL could be an intermediate indicator between many major diseases and mortality. Also, SNHL itself might increase disability and the risks for cognition impairment, accident, depression, or stroke in the elderly (11–13). So, we treated SNHL as an independent variable during analysis with adjustment of other confounding factors for this study. By doing this, we could see whether SNHL could increase the risk of all-cause mortality independently or not.

All above analysis were performed in SAS (version 9.4; SAS Institute, Inc., Cary, NC, USA) and SPSS (version 20.0; IBM Corp., New York, NY, USA). The *p* < 0.05 was considered as having statistically significance.

## 3 Results

Table 1 showed the mean age was 62.6 years [standard deviation (SD) = 5.9] in normal hearing group, 66.6 years (SD = 7.0) in mild SNHL group, and 71.5 (SD = 7.3) in moderate and severe SNHL group. The prevalence of age (*p* < 0.0001), gender (*p* = 0.0320), CAD (*p* = 0.0424), but not other clinical variables, were significantly different between three cohorts.

**Table 1:** Basic characteristics for three groups.

Mean (SD) or *N* (%)	Normal group (*n* = 94)	Mild hearing-impaired group (*n* = 111)	Moderate to severe hearing-impaired group (*n* = 113)	*p* value^*^
Age, years	62.6 (5.9)	66.6 (7.0)	71.5 (7.3)	<0.0001
Female/male, %	68.1/31.9	55.0/45.1	50.4/49.6	0.0320
BMI, kg/m^2^	24.4 (3.5)	24.7 (3.6)	24.2 (3.9)	0.5329
Hypertension, %	25.5	32.4	36.3	0.2501
Diabetes mellitus, %	12.8	15.3	15.9	0.7999
Dyslipidemia, %	5.3	6.3	9.7	0.4255
CAD, %	0	0.9	4.4	0.0424
AMI, %	0	0	0	–
CKD, %	6.4	4.5	3.5	0.6250
Hepatitis, %	11.7	12.6	7.1	0.3508
Cirrhosis, %	2.1	0	2.7	0.2446
Stroke, %	2.1	3.6	2.7	0.8095
Alzheimer’s disease, %	1.1	0	0	0.3026
Parkinson’s disease, %	0	0	0	–
Trauma, %	2.1	2.7	3.5	0.8261
Cancers, %	5.3	3.6	4.4	0.8369
Death, %	4.3	6.3	18.6	<0.0001

AMI, acute myocardial infarction; BMI, body mass index; CAD, coronary artery disease; CKD, chronic kidney disease; SD, standard deviation.

^*^p values were acquired by Pearson’s chi-squared test or one-way ANOVA test for categorical or continuous variables, respectively.

The mean duration of follow-up was 8.54 years for normal hearing group, 8.33 years for mild SNHL group, and 8.25 years for moderate and severe SNHL group (*p* = 0.214). The incidence rates of mortality were 0.04 per 10^2^ person-years of follow-up in normal hearing group, 0.06 per 10^2^ person-years of follow-up in mild SNHL group, and 0.19 per 10^2^ person-years of follow-up in moderate and severe SNHL group, respectively (*p* = 0.0008).

**Figure 1:**
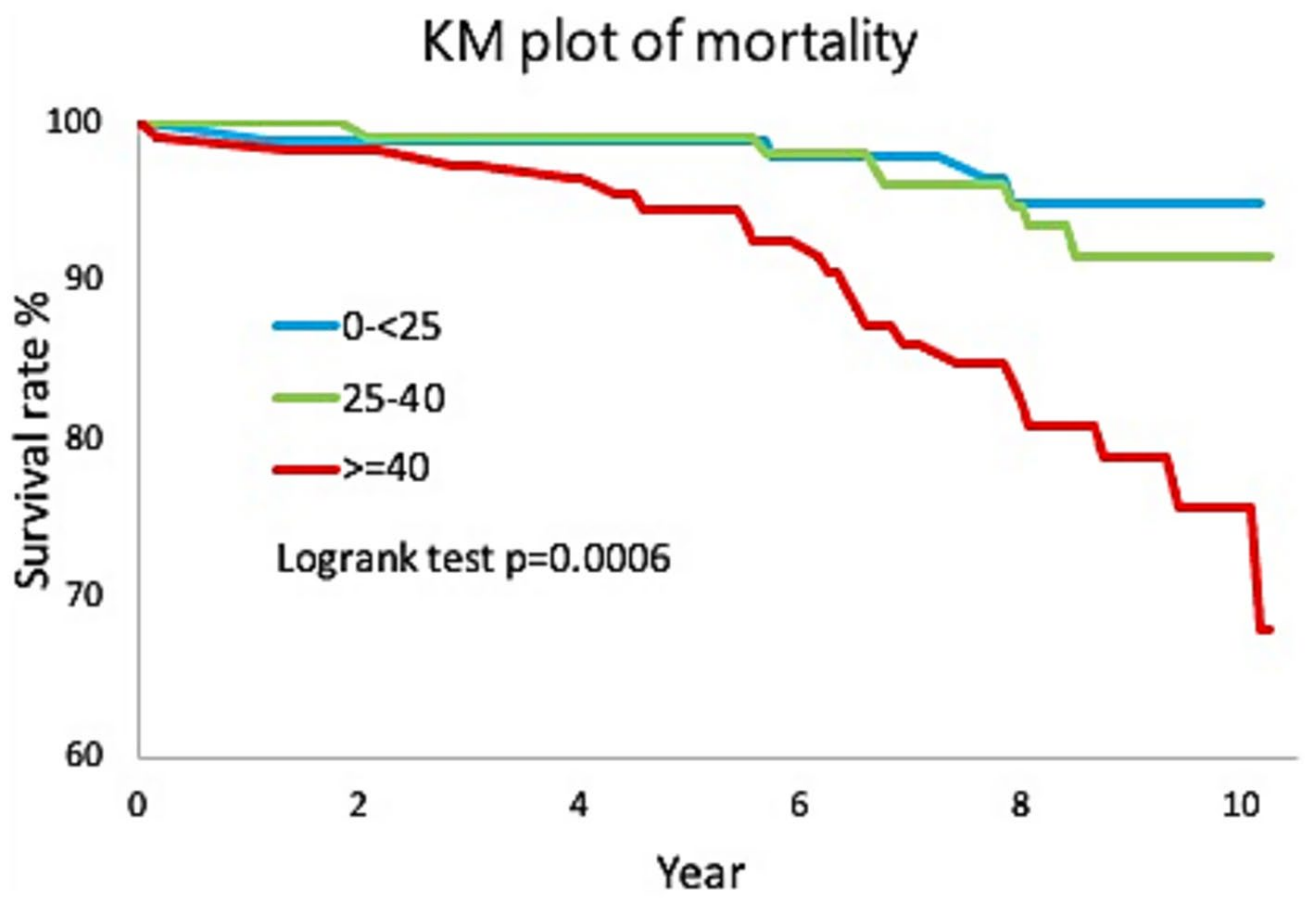
The survival rates in three groups. The survival rate in moderate and severe SNHL group, but not in mild SNHL group, was significantly lower than that in normal hearing group (log rank, *p* = 0.0006).

Figure 1 showed the survival rates in three groups. The survival rate in moderate and severe SNHL group, but not in mild SNHL group, was significantly lower than that in normal hearing group (log rank, *p* = 0.0006). In other words, the elderly with moderate and severe SNHL have a significantly higher mortality rate than those with normal hearing.

Compared to the normal hearing group, the crude HR and aHR for mortality in mild SNHL group was 1.51 (95% CI = 0.44–5.14, *p* = 0.5143) and 2.14 (95% CI = 0.45–10.23, *p* = 0.3396), respectively. And, the crude HR and aHR for mortality in moderate and severe SNHL group was 4.82 (95% CI = 1.65–14.07, *p* = 0.0041) and 6.96 (95% CI = 1.60–30.23, *p* = 0.0096), respectively.

## 4 Discussion

In this cohort study based on detailed audiometry and clinical data, we have considered many clinical variables which might be associated with increased mortality during statistical analysis. The main finding was that the elderly with moderate and severe SNHL of age between 51 and 88 years are at greater risk of mortality than those with normal hearing significantly and independently in Taiwan with relatively low genetic heterogeneity (5, 6).

Comparison with previous literatures, our results were slightly different to previous studies. In our study, moderate and severe SNHL was significantly associated with mortality. In Kim’s study (2020), severe and profound SNHL was significantly associated with mortality in Korean older adults (1). In Fang et al.’s (2) and Cui and Yan’s (3) studies, all degrees of SNHL were associated with mortality in adult Americans (2, 3). However, there was no association between SNHL and mortality in Zhang et al.’s (4) study in Chinese older people. These inconsistency between previous studies might be caused by different study population, imperfect classification of hearing impairment, and/or statistical strategy.

In addition, the SNHL-related mortality risk could be interacted by family status and visual acuity. Divorced status raised the adjusted SNHL-related mortality risk in subjects below 75 years old in Norway (16). Comparing with subjects without visual and hearing impairment, those with both of visual and hearing impairment have a higher risk for mortality (aHR = 1.21). Those with visual impairment only have a slightly elevated risk for mortality (aHR = 1.10), but those with hearing impairment only were not significantly associated with increased mortality in subjects aged 80 and older, adjusting for socio-demographic status, life style, and common diseases (4).

In the aspect of biological plausibility, we believed that oxidative stress and neural inflammation are the most important mechanisms for SNHL in older adults (17–21). So, we suggested that SNHL was not only as an intermediate indicator between many major diseases and increased mortality, but also played an independent role on the mortality in older adults.

However, this study has several limitations. First, severity, duration and treatment compliance of major common diseases were not measured. Second, habits, exercise, socioeconomic status, geographic region, or urbanization level were unmeasured, too. However, we supposed that these confounder factors might affect three cohorts equally. So, these biases would not violate the results significantly.

## 5 Conclusion

Taken together with the findings of previous studies and ours, we suggested that SNHL, especially of moderate to severe or profound severity, was associated with increased risks of mortality in the elderly independently in Taiwan. Therefore, we clinicians should pay more attention to SNHL. To treat its underlying risk factors and/or comorbidity. Also, we should encourage our hearing-impaired patients to use hearing aids more aggressively. By doing so, we could reduce the mortality rate in the elderly.

## Data Availability

The original contributions presented in the study are included in the article/supplementary material, further inquiries can be directed to the corresponding authors.
